# Analytical Modelling of Orthotropic Transient Heat Conduction in the Thermal Therapy Mask Within the Symplectic Framework

**DOI:** 10.3390/mi16111277

**Published:** 2025-11-13

**Authors:** Jinbao Li, Dian Xu, Chengjie Guo, Zhishan Chen, Linchi Jiang, Rui Li

**Affiliations:** School of Mechanics and Aerospace Engineering, State Key Laboratory of Structural Analysis, Optimization and CAE Software for Industrial Equipment, and International Research Center for Computational Mechanics, Dalian University of Technology, Dalian 116024, China; jinbao_li@mail.dlut.edu.cn (J.L.); 1784971935@mail.dlut.edu.cn (C.G.); 1164914977@mail.dlut.edu.cn (Z.C.); 2309853766@mail.dlut.edu.cn (L.J.); ruili@dlut.edu.cn (R.L.)

**Keywords:** orthotropic transient heat conduction, symplectic superposition method, analytical modelling, Robin boundary

## Abstract

The thermal therapy mask, as a wearable device, requires precise thermal management to ensure therapeutic efficacy and safety, which necessitates a detailed investigation of its heat conduction behavior under complex conditions. However, the heat convective behavior of an orthotropic thermal therapy mask with an embedded line heat source under practical operational conditions has not yet been rigorously investigated. Therefore, this study addresses this specific problem by abstracting it into a 2D orthotropic transient heat conduction problem with a line heat source under Robin BCs, and derives its analytical solution using the SSM without any assumption of solution form. The SSM first transforms the governing equation into the frequency domain via the Laplace transform technique and reformulates it within the Hamiltonian framework. The original problem is then decomposed into two subproblems, which are solved by the method of separation of variables and the symplectic eigen expansion. The final analytical solution is obtained through superposing the solutions of the subproblems, and its accuracy is validated through comparison with the finite element method. The influence of the heat convection coefficient on the thermal behavior is systematically analyzed, revealing that increasing the heat convection coefficient accelerates the procedure from transient to steady state and results in reduced steady-state temperature. Furthermore, the analysis of orthotropic thermal conductivity reveals a “short-plank effect”, where the temperature evolution is limited by the smaller thermal conductivity. This study provides benchmark results for accurate and efficient thermal prediction and may enable an extension to broader applications in flexible electronics such as wearable sensors and displays.

## 1. Introduction

Flexible electronics have revolutionized wearable technologies, enabling advanced applications in healthcare [1], therapeutics [2], personal care [3], etc. These applications range from advanced monitoring for personalized healthcare, using technologies like wearable biosensors [4] and multifunctional hydrogels [5], to active therapeutic devices. The thermal therapy mask [6] has gained prominence for its ability to deliver localized and controlled heat, facilitating benefits such as enhanced blood circulation, skin rejuvenation, and pain relief. These devices incorporate flexible heating elements, such as planar or filament-based heat sources, embedded within soft, conformable substrates to ensure user comfort and adaptability to complex geometries. Recent progress in materials science and microfabrication has significantly advanced the development of such devices, offering new opportunities for tailored thermal therapy solutions [7,8]. Despite these advancements, analytical modeling and prediction of heat conduction in the thermal therapy mask remain challenging. This is primarily due to the mathematical difficulties posed by material orthotropy and complex boundary conditions (BCs) within a high-order partial differential equation (PDE). Orthotropy introduces additional mathematical complexity into the governing PDE, making analytical solutions difficult to obtain. This property is characterized by distinct thermal conductivities in the parallel and perpendicular directions of the mask’s 2D rectangular cross-section. Additionally, Robin BCs [9], which account for convective heat exchange with the environment, introduce more complexity compared to simpler Dirichlet or Neumann BCs [10]. These factors collectively increase the mathematical intricacy of solving the high-order PDE, requiring the development of advanced computational approaches to accurately predict the mask’s thermal behavior.

**Figure 1:**
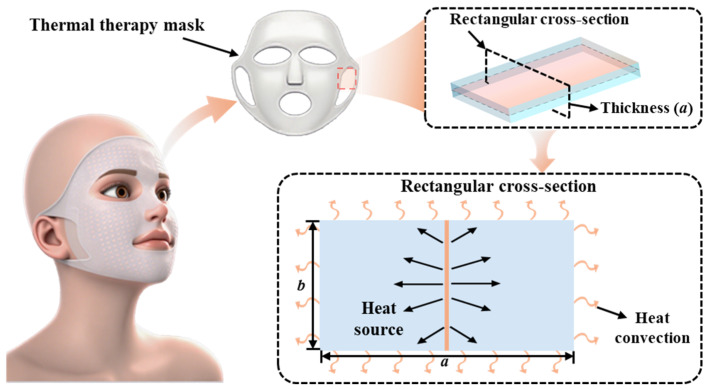
Schematic evolution of a thermal therapy mask into a mathematical model. The modelling process of the thermal therapy mask’s 3D physical structure into a 2D problem is shown, with simplification of the internal planar heat source to a line source and description of boundary heat exchanges by Robin BCs.

Figure 1 presents the progression from a thermal therapy mask’s physical structure to a 2D orthotropic transient heat conduction problem in a rectangular domain incorporating a heat source and Robin BCs. Specifically, the thermal therapy mask typically incorporates an internal planar heat source. To investigate its heat conduction behavior, a cross-section encompassing this planar heat source is selected for analysis. The original planar heat source is simplified into a line heat source within the 2D model, while the heat exchange at the rectangular edges is uniformly represented as generalized convection using Robin BCs. This type of BC effectively captures various forms of heat exchange, whether convective exchange between the mask and the skin or air, or conductive heat transfer from the mask’s cross-section to other parts. This simplification is well-justified as it accurately models the critical physical process of heat conduction [11], while reducing model complexity and computational costs. It should be noted that the present 2D cross-section model, while capturing the non-uniform temperature within the thickness plane, is a local analysis. Its primary limitation is the assumption of thermal uniformity in the third dimension. This idealization, along with the line source simplification, neglects global spatial non-uniformities. Future work should therefore focus on 3D heat conduction analysis.

The thermal therapy mask system, depicted in Figure 1, can be characterized by a temperature field distribution that varies both spatially and temporally. Given the challenges associated with orthotropy and Robin BCs, various methods have been employed to address heat conduction challenges in different systems. Among these, numerical methods are widely adopted due to their flexibility in handling complex geometries and BCs. For instance, Li et al. [12] developed an advanced temporal finite element method (FEM) to tackle both Fourier and non-Fourier heat conduction problems. Sun et al. [13] applied the finite difference method to simulate temperature profiles in coated tools during sustainable machining processes. Li et al. [14] utilized the dual reciprocity method to convert boundary-domain integral equations into boundary integral equations for heat conduction involving internal heat sources. Zhou et al. [15] adopted the polygonal boundary element method to solve transient heat conduction problems with spatially varying heat generation. Furthermore, researchers have explored the meshless method [16], the boundary mapped collocation method [17], and the collocation method [18] to address heat conduction problems involving complex geometries and BCs. However, the unique value of the analytical model remains significant, as it provides explicit mathematical relationships that offer intuitive insights into the relationships between material parameters and thermal behavior, which are critical for optimizing device design. Yin et al. [19] formulated a comprehensive analytical model using the Hankel transform to investigate the thermal characteristics of exothermic flexible electronics with intricate heat source geometries. He et al. [20] employed the method of separation of variables to solve for the orthotropic transient heat conduction in stretchable rectangular heat sources, examining heat flux control and temporal temperature homogenization. Jain and Krishnan [21] leveraged Heaviside functions to precisely represent discrete thermal property distributions, enabling solutions for complex geometries. Building on these established analytical methods, new approaches continue to be developed to further enhance the understanding of these complex transient heat conduction scenarios.

The novel symplectic superposition method (SSM) has recently been proposed by integrating the symplectic framework [22] with the principle of superposition, offering a versatile analytical method that does not require predefined solution forms. This method has been successfully extended from its origins in structural mechanics [23,24,25] to applications in transient heat conduction [10,11]. Although existing analytical studies have solved certain aspects, the heat convective behavior of an orthotropic thermal therapy mask with an embedded line source has not been investigated. To analyze this behavior, the physical system is abstracted into a 2D orthotropic transient heat conduction problem with a line heat source under Robin BCs. This study applies the SSM to derive an analytical solution for this specific problem without any assumption of solution form, providing a new analytical benchmark for the thermal behavior of masks under realistic heat exchange conditions. There is a key knowledge gap in the solution procedure that directly addresses the implicit eigenvalue problem that arises from the Robin BCs, rather than requiring boundary constructions to convert it into an explicit problem. The SSM not only serves as a pivotal tool for parametric analyses and optimization, but also holds future potential for extension to more complex problems, such as 3D analysis, heat conduction in multilayer devices, or scenarios involving radiative heat conduction.

The structure of this paper is as follows. The governing equation of 2D orthotropic transient heat conduction is introduced into the symplectic framework in Section 2. Section 3 presents the solution procedure for subproblems, with the final solution derived through superposition. In Section 4, a convergence analysis of the analytical solution is conducted, and its validity and accuracy are verified by comparison with the FEM results. Furthermore, a parametric study of the heat convection coefficient is performed to assess its impact on temperature changes over time. The conclusions are summarized in Section 5.

## 2. Governing Equation of 2D Orthotropic Transient Heat Conduction Within the Symplectic Framework

**Figure 2:**
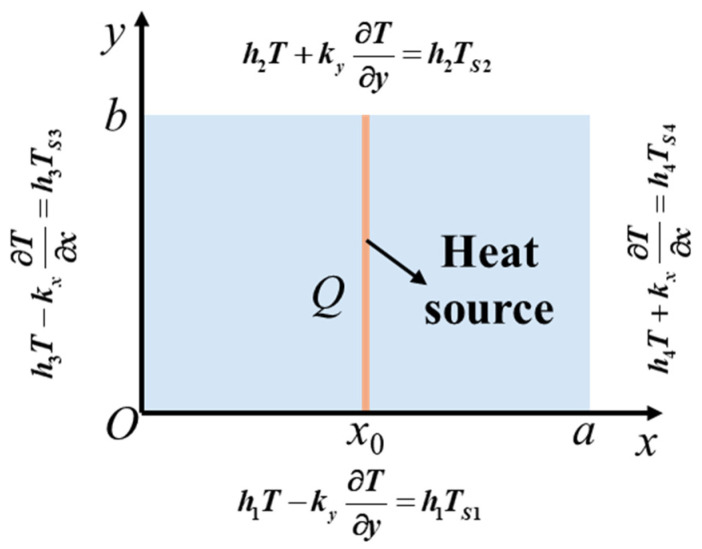
Schematic of an orthotropic rectangular domain with a line heat source. The model dimensions, location of the heat source, coordinate system, and BCs are indicated.

Figure 2 schematically illustrates the 2D orthotropic transient heat conduction model of the thermal therapy mask. The lower-left corner of the orthotropic rectangular domain is placed at the origin *O*, whose length along the Ox axis is *a* and width along the Oy axis is *b*. A line heat source of intensity *Q* perpendicular to the Ox axis is located at $x=x_{0}$. The governing equation for the 2D transient heat conduction is given by [26]

(1) $$k_{x}\frac{\partial^{2}T}{\partial x^{2}}+k_{y}\frac{\partial^{2}T}{\partial y^{2}}+Q\delta \left(x-x_{0}\right)=c\rho \frac{\partial T}{\partial t}$$

where T is temperature, $k_{x}$ and $k_{y}$ represents the thermal conductivity along the Ox and Oy axis, respectively, c is the specific heat capacity, ρ is the density, t is time, Q is the heat source strength, and $\delta \left(x-x_{0}\right)$ is the Dirac delta function that satisfies $\delta \left(x-x_{0}\right)=0$ for $x\neq x_{0}$ and $\int_{-\infty }^{\infty }\delta \left(x-x_{0}\right)dx=1$ for $x=x_{0}$. The domain is subjected to Robin BCs at all four edges, expressed as

(2) $$\begin{array}{l} h_{i}T+k_{y}\frac{\partial T}{\partial n_{y}}=h_{i}T_{Si}\left(i=1,2\right)\\ h_{j}T+k_{x}\frac{\partial T}{\partial n_{x}}=h_{j}T_{Sj}\left(j=3,4\right) \end{array}$$

where $n_{x}$ and $n_{y}$ are the normal vectors in the Ox or Oy direction, $h_{i}$ and $h_{j}$ are the heat convection coefficients, and $T_{Si}$ and $T_{Sj}$ represent the ambient temperatures.

To solve the governing equation, the Laplace transform [27] is applied to the time-dependent function $f\left(t\right)$, defined on $\left[0,+\infty \right)$, as follows

(3) $$F\left(s\right)=\int_{0}^{+\infty }f\left(t\right)e^{-st}dt$$

where s=σ+Iτ is the frequency domain parameter with the imaginary unit I. The corresponding inverse transformation and the first order differential property are

(4) $$\begin{array}{l} f\left(t\right)=\frac{1}{2\pi I}\underset{\tau \to \infty }{\lim}\int_{\sigma -I\tau }^{\sigma +I\tau }e^{st}F\left(s\right)\;ds\\ L\left[\frac{\partial f\left(t\right)}{\partial t}\right]=sL\left[f\left(t\right)\right]-f\left(0\right) \end{array}$$

Therefore, given the initial condition $T\left(x,y,0\right)=T_{0}$, the governing equation in the frequency domain is

(5) $$k_{x}\frac{\partial^{2}\bar{T}\left(x,y,s\right)}{\partial x^{2}}+k_{y}\frac{\partial^{2}\bar{T}\left(x,y,s\right)}{\partial y^{2}}+Q\delta \left(x-x_{0}\right)=c\rho \left[s\bar{T}\left(x,y,s\right)-T_{0}\right]$$

where the variables mentioned with an overbar denote those in the frequency domain. Define $\overset{\cdot }{\left(\right)}=\partial /\partial x$, and the corresponding Lagrangian density function is

(6) $$L=\frac{1}{2}k_{x}{\dot{\bar{T}}}^{2}+\frac{1}{2}k_{y}{\left(\frac{\partial \bar{T}}{\partial y}\right)}^{2}+\frac{c\rho \bar{T}\left(s\bar{T}-T_{0}\right)}{2}-\bar{T}Q\delta \left(x-x_{0}\right)$$

Let $\xi =\bar{T}$, then the corresponding dual variable is

(7) $$\zeta =\frac{\partial L}{\partial \dot{\xi }}=k_{x}\dot{\xi }$$

Furthermore, the Hamiltonian density function is

(8) $$H\left(\xi ,\zeta \right)=\zeta \dot{\xi }-L=\frac{\zeta^{2}}{2k_{x}}-\frac{k_{y}}{2}{\left(\frac{\partial \xi }{\partial y}\right)}^{2}-\frac{c\rho \xi \left(s\xi -T_{0}\right)}{2}+\xi Q\delta \left(x-x_{0}\right)$$

By taking the variation of Equation (8), we obtain

(9) $$\begin{array}{l} \frac{\partial H}{\partial \zeta }=\frac{\zeta }{k_{x}}\\ \frac{\partial H}{\partial \xi }=-k_{y}\frac{\partial^{2}\xi }{\partial y^{2}}-cs\rho \xi +\frac{c\rho T_{0}}{2}+Q\delta \left(x-x_{0}\right) \end{array}$$

Substituting Equation (9) into the Hamiltonian canonical equation $\dot{\xi }=\partial H/\partial \zeta$ and $\dot{\zeta }=-\partial H/\partial \xi$, the matrix-form Hamiltonian-system equation can be obtained

(10) $$\frac{\partial Z}{\partial x}=HZ+f$$

where $Z={\left[\xi ,\zeta \right]}^{T}$, $f={\left[0,-Q\delta \left(x-x_{0}\right)-c\rho T_{0}/2\right]}^{T}$, and $H=\left[\begin{array}{cc} 0 & 1/k_{x}\\ -k_{y}\partial^{2}/\partial y^{2}+cs\rho & 0 \end{array}\right]$ satisfying $H^{T}=JHJ$. Here, $J=\left[\begin{array}{cc} 0 & I\\ -I & 0 \end{array}\right]$ is the symplectic matrix [22] satisfying $J=-J^{-1}$. The Hamiltonian framework can be readily extended to non-uniform initial value problems. This is because any arbitrary initial conditions are naturally incorporated into Equation (10) as an inhomogeneous term. Here, uniform zero initial conditions are assumed for simplicity. Applying the method of separation of variables to the homogeneous equation of Equation (10), by setting $Z\left(x,y\right)=Y\left(y\right)X\left(x\right)$, yields an eigenvalue equation and an ordinary differential equation

(11) $$\begin{array}{l} HY\left(y\right)=\mu Y\left(y\right)\\ \frac{dX\left(x\right)}{dx}=\mu X\left(x\right) \end{array}$$

where $Y\left(y\right)={\left[\xi \left(y\right),\eta \left(y\right)\right]}^{T}$ is the eigenvector, and μ is the associated eigenvalue. Evidently, −μ also qualifies as an eigenvalue [11]. As a result, the eigenvalues amount to 2*n* $\left(n=1,2,3,\cdots \right)$ in total, given by $\mu_{n}$ and $-\mu_{n}$. Furthermore, the characteristic equation of the first part of Equation (11) can be obtained

(12) $$\mu^{2}-\frac{k_{y}\lambda^{2}}{k_{x}}+\frac{cs\rho }{k_{x}}=0$$

with the characteristic roots $\lambda_{1,2}=\pm I\beta$ and $\beta =\sqrt{k_{x}\mu^{2}-cs\rho }$. Therefore, the general solution of the temperature field is

(13) $$\bar{T}\left(y\right)=C_{1}\cos\left(\beta y\right)+C_{2}\sin\left(\beta y\right)$$

where $C_{1}$ and $C_{2}$ are coefficients to be determined.

## 3. Solution Procedure of 2D Orthotropic Transient Heat Conduction Within Symplectic Framework

**Figure 3:**
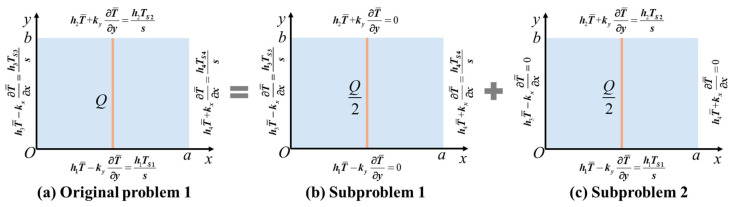
Decomposition of an orthotropic rectangular domain under Robin BCs. The original problem (**a**) shows a rectangular domain with a line heat source and under various Robin BCs at four edges. Subproblem 1 (**b**) depicts the same domain with a line heat source Q/2 and under homogeneous Robin BCs at y=0 and y=b. Subproblem 2 (**c**) depicts the same domain with a line heat source Q/2 and under homogeneous Robin BCs at x=0 and x=a.

To solve the 2D orthotropic transient heat conduction problem with a line heat source under Robin BCs, as depicted in Figure 3, the SSM is employed within the Hamiltonian framework to derive the analytical solution. The original problem (Figure 3a) is decomposed into subproblem 1 (Figure 3b) and subproblem 2 (Figure 3c), each of which features homogeneous Robin BCs on a pair of opposite edges. Taking subproblem 1 as an example, the homogeneous BCs at edges y=0 and y=b are

(14) $$\begin{array}{l} {\left.\left(h_{1}\bar{T}-k_{y}\frac{\partial \bar{T}}{\partial y}\right)\right|}_{y=0}=0\\ {\left.\left(h_{2}\bar{T}+k_{y}\frac{\partial \bar{T}}{\partial y}\right)\right|}_{y=b}=0 \end{array}$$

Substituting Equation (13) into Equation (14), the nontrivial solution of Equation (13) requires that

(15) $$\left(h_{1}+h_{2}\right)k_{y}\beta \cos\left(\beta b\right)+\left(h_{1}h_{2}-k_{y}^{2}\beta^{2}\right)\sin\left(\beta b\right)=0$$

from which we can obtain an infinite number of eigenvalues β. The *n*-th term $\beta_{n}$ can be uniquely determined from Equation (15). Consequently, the symplectic eigenvector can be identified as

(16) $$Y\left(y\right)=\left[Y_{1}\left(y\right),Y_{-1}\left(y\right),Y_{2}\left(y\right),Y_{-2}\left(y\right),\cdots ,Y_{n}\left(y\right),Y_{-n}\left(y\right),\cdots \right]$$

where $Y_{\pm n}\left(y\right)=\left[\cos\left(\beta_{n}y\right)+\phi_{n}\sin\left(\beta_{n}y\right)\right]{\left[1,\pm k_{x}\mu_{n}\right]}^{T}$, $\mu_{n}=\sqrt{\left(k_{y}\beta_{n}^{2}+sc\rho \right)/k_{x}}$ and $\phi_{n}=h_{1}/\left(k_{y}\beta_{n}\right)$$\left(n=1,2,3,\cdots \right)$. $Y_{n}\left(y\right)$ and $Y_{-n}\left(y\right)$ satisfy the symplectic conjugate orthogonality relation. Accordingly, the state vector can be first expressed in terms of the eigenvectors as $Z\left(x,y\right)=Y\left(y\right)X\left(x\right)$. The nonhomogeneous term f is expanded similarly, as $f=Y\left(y\right)F$. With the relation $HY\left(y\right)=Y\left(y\right)U$ with $U=\mathrm{diag}\left[\mu_{1},\mu_{-1},\mu_{2},\mu_{-2},\cdots ,\mu_{n},\mu_{-n},\cdots \right]$, Equation (10) reduces to

(17) $$\frac{dX\left(x\right)}{dx}=UX\left(x\right)+F$$

where $X\left(x\right)={\left[X_{1}\left(x\right),X_{-1}\left(x\right),X_{2}\left(x\right),X_{-2}\left(x\right),\cdots X_{n}\left(x\right),X_{-n}\left(x\right),\cdots \right]}^{T}$ and $F={\left[F_{1},F_{-1},F_{2},F_{-2},\cdots ,F_{n},F_{-n},\cdots \right]}^{T}$. The vector F can be obtained by left-multiplying both sides of $f=Y\left(y\right)F$ by $Y{\left(y\right)}^{T}J$ and integrating over $y\in \left[0,b\right]$, with the components written as

(18) $$F_{-n}=-F_{n}=Q\delta \left(x-x_{0}\right)\tau_{n}\left\{\phi_{n}\cos\left(b\beta_{n}\right)-\left[\phi_{n}+\sin\left(b\beta_{n}\right)\right]\right\}$$

where $\tau_{n}=1/s\mu_{n}k_{x}\left\{2\left[\phi_{n}+b\left(1+\phi_{n}^{2}\right)\beta_{n}\right]-2\phi_{n}\cos\left(2b\beta_{n}\right)-\left(\phi_{n}^{2}-1\right)\sin\left(2b\beta_{n}\right)\right\}$. The general solution of the homogeneous equation for Equation (17) can be written as

(19) $$X\left(x\right)=\Phi \left(x\right)A$$

where $A={\left[A_{1},B_{1},A_{2},B_{2},\cdots ,A_{n},B_{n},\cdots \right]}^{T}$ and $\Phi \left(x\right)=e^{Ux}$. The solution of the nonhomogeneous equation is further obtained

(20) $$X\left(x\right)=\Phi \left(x\right)A+\Phi \left(x\right){\int_{0}^{x}\Phi \left(r\right)}^{-1}F\left(r\right)dr$$

Therefore, the specific solution $X_{\pm 1n}\left(x\right)$ can be expressed as

(21) $$\begin{array}{l} X_{n}=e^{\mu_{n}x}\left\{A_{n}+QH\left(x-x_{0}\right)\tau_{n}e^{-x_{0}\mu_{n}}\left\{\phi_{n}\left[\cos\left(b\beta_{n}\right)-1\right]-\sin\left(b\beta_{n}\right)\right\}\right\}\\ X_{-n}=e^{-\mu_{n}x}\left\{B_{n}-QH\left(x-x_{0}\right)\tau_{n}e^{x_{0}\mu_{n}}\left\{\phi_{n}\left[\cos\left(b\beta_{n}\right)-1\right]-\sin\left(b\beta_{n}\right)\right\}\right\} \end{array}$$

where $H\left(x-x_{0}\right)$ is the Heaviside function [28] that satisfies $H\left(x-x_{0}\right)=0$ for $x<x_{0}$ and $H\left(x-x_{0}\right)=1$ for $x\geq x_{0}$. The temperature field of subproblem 1 of the original problem is expressed as

(22) $$\begin{array}{c} {\bar{T}}_{1}\left(x,y,s\right)=\sum_{n=1}^{\infty }\left[\cos\left(\beta_{n}y\right)+\phi_{n}\sin\left(\beta_{n}y\right)\right]\left\{A_{n}e^{\mu_{n}x}+B_{n}e^{-\mu_{n}x}+Q\tau_{n}H\left(x-x_{0}\right)\right.\\ \;\times \left.\left[e^{\mu_{n}\left(x-x_{0}\right)}-e^{-\mu_{n}\left(x-x_{0}\right)}\right]\left\{\phi_{n}\left[\cos\left(b\beta n\right)-1\right]-\sin\left(b\beta_{n}\right)\right\}\right\} \end{array}$$

where $A_{n}$ and $B_{n}$ are undetermined hitherto. By expanding the BCs at x=0 and x=a in terms of the symplectic eigenvectors and substituting them into Equation (22), the analytical solution for the temperature field in subproblem 1 is obtained

(23) $$\begin{array}{cc} {\bar{T}}_{1}\left(x,y,s\right) & =-2\sum_{n=1,2,3,\cdots }^{\infty }\frac{\tau_{n}\left[\cos\left(\beta_{n}y\right)+\phi_{n}\sin\left(\beta_{n}y\right)\right]\left\{\phi_{n}\left[\cos\left(b\beta_{n}\right)-1\right]-\sin\left(b\beta_{n}\right)\right\}}{k_{x}\mu_{n}\left(h_{3}+h_{4}\right)\cosh\left(a\mu_{n}\right)+\left(h_{3}h_{4}+k_{x}^{2}\mu_{n}^{2}\right)\sinh\left(a\mu_{n}\right)}\\ & \times \left\{2h_{3}k_{x}T_{S3}\mu_{n}\left\{k_{x}\mu_{n}\cosh\left[\left(a-x\right)\mu_{n}\right]+h_{4}\sinh\left[\left(a-x\right)\mu_{n}\right]\right\}\right.\\ & +\left\{2h_{4}k_{x}k_{y}T_{S4}\beta_{n}\mu_{n}+Q\left\{k_{x}\mu_{n}\cosh\left[\left(a-x_{0}\right)\mu_{n}\right]+h_{4}\sinh\left[\left(a-x_{0}\right)\mu_{n}\right]\right\}\right\}\\ & \times \left[k_{x}\mu_{n}\cosh\left(\mu_{n}x\right)+h_{3}\sinh\left(\mu_{n}x\right)\right]-QH\left(x-x_{0}\right)\tau_{n}\sinh\left[\left(x-x_{0}\right)\mu_{n}\right]\\ & \left.\times \left[k_{x}\mu_{n}\left(h_{3}+h_{4}\right)\cosh\left(a\mu_{n}\right)+\left(h_{3}h_{4}+k_{x}^{2}\mu_{n}^{2}\right)\sinh\left(a\mu_{n}\right)\right]\right\} \end{array}$$

The solution procedure for subproblem 2 follows a methodology analogous to that of subproblem 1. The eigen equation of subproblem 2 is

(24) $$\left(h_{3}+h_{4}\right)k_{x}\alpha \cos\left(\alpha a\right)+\left(h_{3}h_{4}-k_{x}^{2}\alpha^{2}\right)\sin\left(\alpha a\right)=0$$

And the analytical solution for the temperature field in subproblem 2 is as follows

(25) $$\begin{array}{cc} {\bar{T}}_{2}\left(x,y,s\right) & =2\sum_{n=1,2,3,\cdots }^{\infty }\frac{\sigma_{n}\left[\cos\left(\alpha_{n}x\right)+\varphi_{n}\sin\left(\alpha_{n}x\right)\right]}{k_{y}\nu_{n}\left(h_{1}+h_{2}\right)\cosh\left(b\nu_{n}\right)+\left(h_{1}h_{2}+k_{y}^{2}\nu_{n}^{2}\right)\sinh\left(b\nu_{n}\right)}\\ & \times \left\{Q\alpha_{n}\left[\cos\left(\alpha_{n}x_{0}\right)+\varphi_{n}\sin\left(\alpha_{n}x_{0}\right)\right]\left\{\left(h_{1}+h_{2}\right)k_{y}\nu_{n}\cosh\left(b\nu_{n}\right)\right.\right.\\ & +\left(h_{1}h_{2}+k_{y}^{2}\nu_{n}^{2}\right)\sinh\left(b\nu_{n}\right)-h_{2}\left[k_{y}\nu_{n}\cosh\left(\nu_{n}y\right)+h_{1}\sinh\left(\nu_{n}y\right)\right]\\ & \left.-h_{1}\left\{k_{y}\nu_{n}\cosh\left[\left(b-y\right)\nu_{n}\right]+h_{2}\sinh\left[\left(b-y\right)\nu_{n}\right]\right\}\right\}+2k_{y}\nu_{n}^{2}\\ & \times \left\{\sin\left(a\alpha_{n}\right)+\varphi_{n}\left[1-\cos\left(a\alpha_{n}\right)\right]\right\}\left\{h_{2}T_{S2}\left[k_{y}\nu_{n}\cosh\left(\nu_{n}y\right)+h_{1}\sinh\left(\nu_{n}y\right)\right]\right.\\ & \left.\left.+h_{1}T_{S1}\left\{k_{y}\nu_{n}\cosh\left[\left(b-y\right)\nu_{n}\right]+h_{2}\sinh\left[\left(b-y\right)\nu_{n}\right]\right\}\right\}\right\} \end{array}$$

where $\sigma_{n}=1/k_{y}s\nu_{n}^{2}\left\{2\left[\varphi_{n}+a\alpha_{n}\left(1+\varphi_{n}^{2}\right)\right]-2\varphi_{n}\cos\left(2a\alpha_{n}\right)-\left(\varphi_{n}^{2}-1\right)\sin\left(2a\alpha_{n}\right)\right\}$, $\varphi_{n}=h_{3}/\left(k_{x}\alpha_{n}\right)$, and $\nu_{n}=\sqrt{\left(k_{x}\alpha_{n}^{2}+sc\rho \right)/k_{y}}$. Therefore, the final analytical solution of the original problem 1 can be denoted as $\bar{T}\left(x,y,s\right)={\bar{T}}_{1}\left(x,y,s\right)+{\bar{T}}_{2}\left(x,y,s\right)$ in the s-domain.

## 4. Results and Discussion

A rectangular domain is examined, having a length of 1 cm and a width of 1 cm. The domain has a uniform density of $\rho =1.45\;g/{\mathrm{cm}}^{3}$, a specific heat capacity of $c=1.3\;J/\left(g\cdot {}^{\circ}C\right)$, and anisotropic thermal conductivities of $k_{x}=2\;W/\left(\mathrm{cm}\cdot {}^{\circ}C\right)$ and $k_{y}=1\;W/\left(\mathrm{cm}\cdot {}^{\circ}C\right)$. The heat convection coefficients at the four edges are defined as $h_{1}=h_{2}/2=h_{3}/3=h_{4}/4=0.1\;W/\left({\mathrm{cm}}^{2}\cdot {}^{\circ}C\right)$, with ambient temperatures set to $T_{S1}=T_{S2}=T_{S4}=20\;{}^{\circ}C$ and $T_{S3}=37\;{}^{\circ}C$. A line heat source, located at $x_{0}=a/2$ has an intensity of $Q=30\;W/{\mathrm{cm}}^{2}$. The FEM is implemented in Abaqus utilizing four-node heat conduction elements. Based on the dependence study of Case 1 in Table 1, it is evident that the temperature results stabilize to four significant digits at a mesh size of a/200 and a time increment of 0.005 s. The results are considered independent of further mesh refinement or time step reduction, and thus these settings were adopted for comparison with the analytical solution.

Convergence analysis of the SSM is conducted for two cases at t=15 s: Case 1 (with a line heat source) and Case 2 (without a line heat source). To determine the minimum number of terms required for convergence to four significant digits, four typical points are selected for examination: $P_{1}\left(a/5,b/5\right)$, $P_{2}\left(a/5,4b/5\right)$, $P_{3}\left(4a/5,b/5\right)$, and $P_{4}\left(4a/5,4b/5\right)$. The minimum number of terms (highlighted in bold) is listed in Table 2. Since all points in both cases converge to four significant digits using 20 terms, a consistent criterion of 20 terms is adopted for convergence.

**Figure 4:**
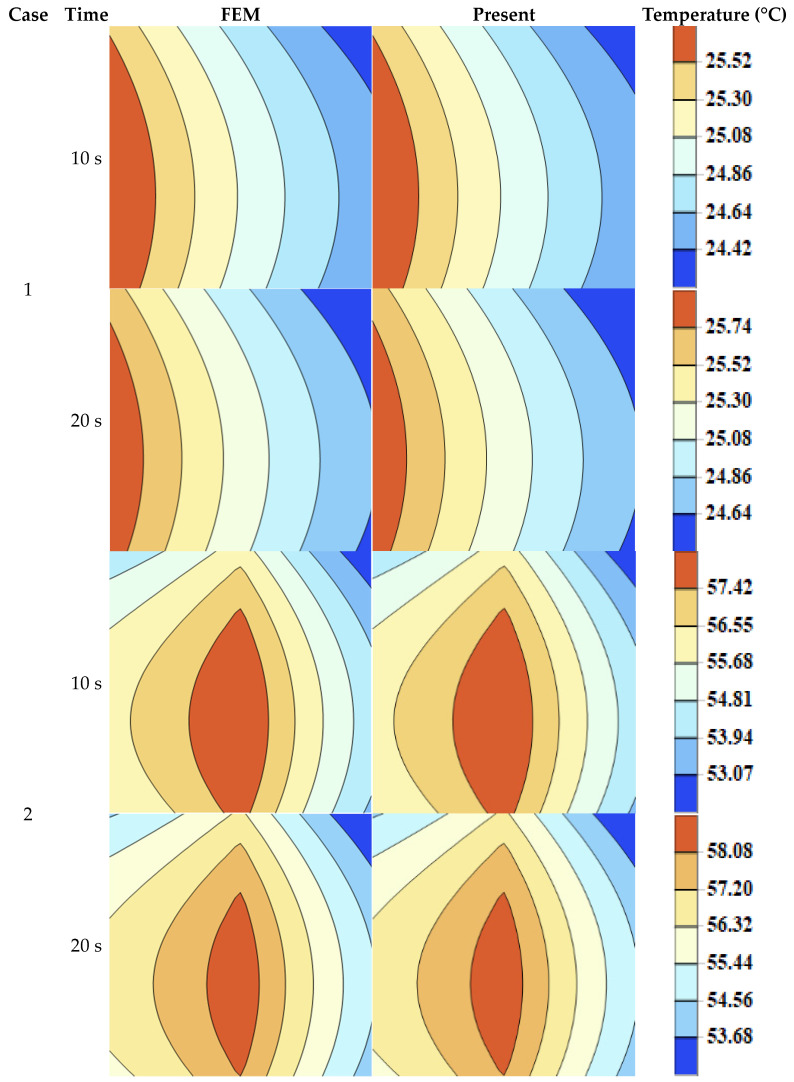
Temperature contour plots at t=10 s and t=20 s for two cases. The SSM and FEM results show good agreement.

To further validate the accuracy of the solution, results are computed at time intervals of 5 s, 10 s, 15 s, 20 s, 25 s, and 30 s and compared with those obtained by the FEM, demonstrating excellent agreement, as summarized in Table 3. Furthermore, temperature contour plots for both cases at 10 s and 20 s are presented by the SSM and FEM, revealing strong consistency in the overall domain, as shown in Figure 4.

**Figure 5:**
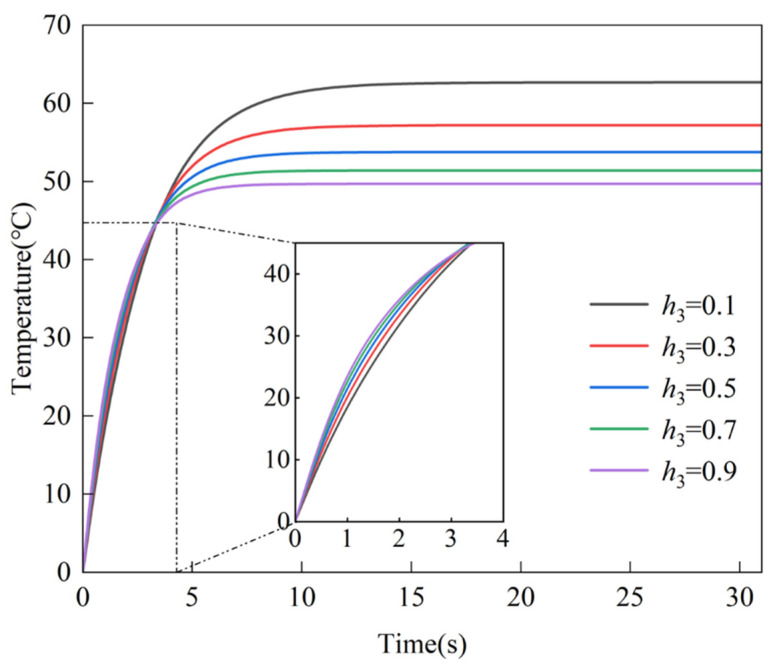
Temperature changes over time at $P_{1}\left(a/5,b/5\right)$ under varying heat convection coefficients at x=0 edge. The curves depict temperature versus time for five coefficients ($h_{3}=$ 0.1, 0.3, 0.5, 0.7, 0.9), color-coded as black, red, blue, green, and purple, respectively.

An analysis of the influence of the heat convection coefficient on the temperature changes over time is conducted. The heat convection coefficient $h_{3}$ at the edge x=0 is varied from 0.1 to 0.9 $W/\left(m^{2}\cdot {}^{\circ}C\right)$, with the resulting temperature changes over time at $P_{1}$ are analyzed, as depicted in Figure 5. A sharp initial rise due to the line heat source at $x_{0}=a/2$ is observed, followed by stabilization, with steady-state temperatures decreasing from approximately 60 °C at $h_{3}=0.1$ to 40 °C at $h_{3}=0.9$. Higher $h_{3}$ values enhance heat dissipation, leading to slower temperature increases and smoother convergence to steady state between 15 s and 20 s. Increased heat convection coefficients $h_{3}$ at the edge x=0 yield a more rapid attainment of thermal steady-state and reduced steady-state temperatures. The parameter sensitivity highlights the critical influence of BCs on thermal dynamics, offering valuable insights for optimizing heat conduction designs, as illustrated in Figure 5. These trends indicate that the heat convection coefficient is a first-order control on both the transient time scale and the final temperature, providing a practical lever for tuning the thermal field in the structural design of such thermal therapy masks.

**Figure 6:**
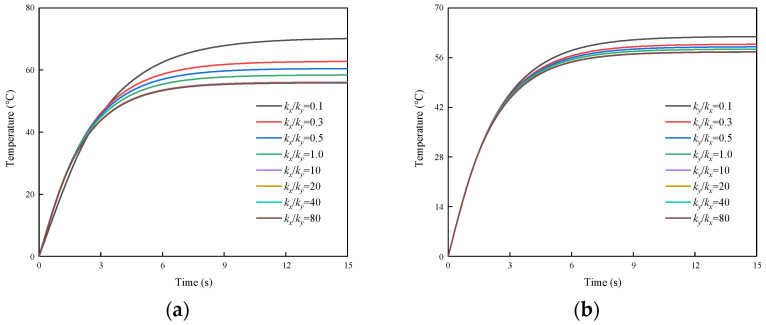
Temperature changes over time at $P_{1}\left(a/5,b/5\right)$ under varying thermal conductivity ratios: (**a**) Temperature versus time for varying $k_{x}/k_{y}$ ratios (0.1, 0.3, 0.5, 1.0, 10, 20, 40, 80) while holding $k_{y}=1.0\;W/\left(\mathrm{cm}\cdot {}^{\circ}C\right)$; (**b**) Temperature versus time for varying $k_{y}/k_{x}$ ratios (0.1, 0.3, 0.5, 1.0, 10, 20, 40, 80) while holding $k_{x}=1.0\;W/\left(\mathrm{cm}\cdot {}^{\circ}C\right)$.

The analysis of the heat conductivities ($k_{x}$ and $k_{y}$) indicates that the temperature variation is governed by a “short-plank effect”, as detailed in Figure 6. A symmetrical behavior is observed in both Figure 6a (holding $k_{y}=1.0\;W/\left(\mathrm{cm}\cdot {}^{\circ}C\right)$) and Figure 6b (holding $k_{x}=1.0\;W/\left(\mathrm{cm}\cdot {}^{\circ}C\right)$): With the increase in one of the thermal conductivities, the steady-state temperature decreases gradually, but the decrease becomes less pronounced. When one thermal conductivity becomes sufficiently greater than the other, the steady-state temperature exhibits only a marginal decrease with further increases in the larger conductivity. This confirms that the smaller thermal conductivity acts as the dominant limiting factor, suppressing the internal heat conduction. Consequently, the heat conduction behavior is dictated by this short plank, and further increases in the larger conductivity component cannot overcome this limitation.

## 5. Conclusions

Accurate thermal management is critical for the efficacy and safety of flexible electronics like the thermal therapy mask. While numerical methods are widely adopted for their flexibility in handling complex geometries [12,13,14,15,16,17], their high computational cost for transient analysis can impede rapid design iteration. On the analytical front, investigations have frequently centered on homogeneous materials [19,20,21] and are often confined to simpler BCs [10], such as Dirichlet or Neumann conditions. However, modeling the thermal therapy mask requires simultaneously addressing material orthotropy and Robin BCs, presenting a more complex analytical challenge that has not been rigorously addressed. This work develops an analytical model for 2D orthotropic transient heat conduction in the thermal therapy mask with a line heat source under Robin BCs. By combining a Laplace-domain transformation with a Hamiltonian system-based formulation, the SSM decomposes the original problem into solvable subproblems whose solutions are recombined to yield a full analytical solution. Cross-validation against the FEM confirms the accuracy of the present approach. This accuracy, combined with the inherent high computational efficiency of an analytical model for parametric analysis and design optimization, makes it a valuable tool for rapid design iteration and real-time thermal prediction in wearable devices. Parametric analysis shows that the edge heat convection coefficient exerts a dominant control on the thermal response: the higher the heat convection coefficient, the faster the procedure from transient to steady state, and the lower the steady-state temperature. This identifies boundary convection as a practical design lever for balancing therapeutic efficacy against skin safety in mask-based thermal therapy. In addition, the orthotropic heat conduction is characterized by a “short-plank effect”, where the smaller thermal conductivity component acts as the rate-limiting factor for heat conduction behavior. The analytical model provides benchmarks for validating numerical solvers and for guiding mask structural design. The scope of the present model is limited by its 2D simplification and the idealization of the internal planar heat source as a line source. Future work could focus on extending this symplectic framework to address 3D analysis, heat transfer in multilayer devices, or scenarios involving radiative heat conduction.

## Figures and Tables

**Table 1:** Mesh size and time increment dependence study of FEM at two representative locations and t=10 s. Temperature solution (°C) from the FEM convergence study, used to validate the selected mesh and time increment parameters.

Location	Mesh Size	Time Increment/s			
		1	0.1	0.01	0.005
$\left(0,2b/5\right)$	a/10	25.55	25.78	25.80	25.80
	a/100	25.55	25.78	25.80	25.80
	a/200	25.55	25.78	25.80	25.80
$\left(a,b\right)$	a/10	24.02	24.23	24.24	24.24
	a/100	24.01	24.22	24.24	24.24
	a/200	24.01	24.22	24.24	24.24

**Table 2:** Convergence analysis of the temperature solution (°C) for cases with and without a heat source at t=15 s**.** The minimum number of terms required for convergence to four significant digits at four representative points is in bold.

Case	Location	Number of Series Terms					
		5	10	15	20	25	30
1	$P_{1}$	**25.60**	25.60	25.60	25.60	25.60	25.60
	$P_{2}$	**25.47**	25.47	25.47	25.47	25.47	25.47
	$P_{3}$	**24.84**	24.84	24.84	24.84	24.84	24.84
	$P_{4}$	**24.71**	24.71	24.71	24.71	24.71	24.71
2	$P_{1}$	57.15	57.19	**57.18**	57.18	57.18	57.18
	$P_{2}$	56.21	56.25	56.25	**56.24**	56.24	56.24
	$P_{3}$	55.97	56.01	**56.00**	56.00	56.00	56.00
	$P_{4}$	55.05	55.09	**55.08**	55.08	55.08	55.08

**Table 3:** Temperature (°C) comparison at representative locations and times. The FEM results are presented for comparison.

Time (s)	Method	Case 1				Case 2			
		$P_{1}$	$P_{2}$	$P_{3}$	$P_{4}$	$P_{1}$	$P_{2}$	$P_{3}$	$P_{4}$
5	Present	23.65	23.57	22.91	22.84	52.80	51.99	51.68	50.88
	FEM	23.66	23.58	22.92	22.84	52.79	51.97	51.66	50.87
10	Present	25.47	25.33	24.70	24.58	55.87	55.94	55.69	54.78
	FEM	25.47	25.34	24.70	24.58	55.86	55.93	55.69	54.78
15	Present	25.60	25.47	24.84	24.71	57.18	56.24	56.00	55.08
	FEM	25.60	25.47	24.84	24.71	57.17	56.24	56.00	55.08
20	Present	25.60	25.47	24.84	24.71	57.20	56.27	56.02	55.10
	FEM	25.62	25.48	24.85	24.72	57.20	56.26	56.02	55.10
25	Present	25.61	25.48	24.85	24.72	57.20	56.27	56.03	55.11
	FEM	25.62	25.48	24.85	24.72	57.20	56.26	56.02	55.10
30	Present	25.61	25.48	24.85	24.72	57.20	56.27	56.03	55.11
	FEM	25.62	25.48	24.85	24.72	57.20	56.26	56.02	55.10

## Data Availability

The Mathematica scripts used to generate the results and figures in this study are available in a public GitHub repository at https://github.com/JINBAOLI-DUT/SSM-THERMAL-THERAPY-MASK, accessed on 4 October 2025.

## Nomenclature

a, b	Length and width along the *Ox* and *Oy* axis direction
$k_{x}$, $k_{y}$	Thermal conductivity along the *Ox* and *Oy* axis direction
T, $T\left(x,y,t\right)$	Temperature in the time domain
$\bar{T}$, $\bar{T}\left(x,y,s\right)$	Temperature in the frequency domain
t	Time
s	Frequency parameters
c	Specific heat capacity
ρ	Density
Q	Heat source strength
$T_{Si}$	Ambient temperature
$h_{i}$	Heat convection coefficient
Z, $Z\left(x,y\right)$	State vector matrix
H	Hamiltonian operator matrix
J	Symplectic matrix
$Y\left(y\right)$	Eigenvector matrix
$\mu_{i}$	Eigenvalue
$\beta_{i}$	Characteristic root
